# Biosynthesis of catechol melanin from glycerol employing metabolically engineered *Escherichia coli*

**DOI:** 10.1186/s12934-016-0561-0

**Published:** 2016-09-22

**Authors:** Alejandra Mejía-Caballero, Ramón de Anda, Georgina Hernández-Chávez, Simone Rogg, Alfredo Martinez, Francisco Bolívar, Victor M. Castaño, Guillermo Gosset

**Affiliations:** 1 Departamento de Ingeniería Celular y Biocatálisis, Instituto de Biotecnología, Universidad Nacional Autónoma de México, Apdo. Postal 510-3, Cuernavaca, MOR CP 62271 Mexico; 2 Centro de Física Aplicada y Tecnología Avanzada, Universidad Nacional Autónoma de México, Santiago de Querétaro, Mexico

**Keywords:** Metabolic engineering, Melanin, Catechol, Tyrosinase, *Escherichia coli*

## Abstract

**Background:**

Melanins comprise a chemically-diverse group of polymeric pigments whose function is related to protection against physical and chemical stress factors. These polymers have current and potential applications in the chemical, medical, electronics and materials industries. The biotechnological production of melanins offers the possibility of obtaining these pigments in pure form and relatively low cost. In this study, *Escherichia coli* strains were engineered to evaluate the production of melanin from supplemented catechol or from glycerol-derived catechol produced by an *Escherichia coli* strain generated by metabolic engineering.

**Results:**

It was determined that an improved mutant version of the tyrosinase from *Rhizobium etli* (MutmelA), could employ catechol as a substrate to generate melanin. Strain *E. coli* W3110 expressing Mut*melA* was grown in bioreactor batch cultures with catechol supplemented in the medium. Under these conditions, 0.29 g/L of catechol melanin were produced. A strain with the capacity to synthesize catechol melanin from a simple carbon source was generated by integrating the gene Mut*melA* into the chromosome of *E. coli* W3110 *trpD9923*, that has been modified to produce catechol by the expression of genes encoding a feedback inhibition resistant version of 3-deoxy-d-*arabino*-heptulosonate 7-phosphate synthase, transketolase and anthranilate 1,2-dioxygenase from *Pseudomonas aeruginosa* PAO1. In batch cultures with this strain employing complex medium with 40 g/L glycerol as a carbon source, 1.21 g/L of catechol melanin were produced. The melanin was analysed by employing Fourier transform infrared spectroscopy, revealing the expected characteristics for a catechol-derived polymer.

**Conclusions:**

This constitutes the first report of an engineered *E. coli* strain and a fermentation process for producing a catechol melanin from a simple carbon source (glycerol) at gram level, opening the possibility of generating a large quantity of this polymer for its detailed characterization and the development of novel applications.

## Background

Melanins are polymeric pigments found in most biological groups [1]. These compounds have diverse functions, mostly related to stress protection from physical and chemical factors. Melanins result from the oxidative polymerization of phenolic and indolic compounds [2]. Monomer precursors for the various types of natural melanins include l-tyrosine, l-3,4-dihydroxyphenylalanine, homogentisic acid, 1,8-dihydroxynaphthalene, glutaminyl-3,4-dihydroxybenzene and catechol [3, 4]. The first step in the synthesis of melanins involves the enzyme-catalysed oxidation of the precursor monomers into their respective quinones, which undergo further spontaneous oxidation to generate the high molecular weight melanins. Tyrosinase and lacasse have been identified as the enzymes involved in melanogenesis in various microbial species. These are copper-containing oxidoreductases, which can employ as substrates various mono- and polyphenolic compounds [5, 6].

Diverse studies have determined potential industrial applications for melanins. These polymers can act as UV absorbers, scavengers of free radicals and reactive oxygen species, amorphous semiconductors, cation exchangers, X and γ-ray absorbers [2, 7, 8]. Melanins can also serve as a template for the synthesis of silver and gold nanostructures with potential uses in the medical and food industries [9, 10]. Considering the physicochemical complexity and diversity of melanins, it is expected that further industrial applications will be found in the near future.

There is considerable interest in the development of production processes for obtaining melanins with high purity and at a relatively low cost. The biotechnological production of melanin derived from l-tyrosine (eumelanin) has been reported employing recombinant *Escherichia coli* strains. This has been achieved by the heterologous expression of the gene encoding the enzyme tyrosinase from the bacteria *Rhizobium etli* or *Streptomyces antibioticus* [11–13]. The production processes with these recombinant microorganisms are based on the conversion of l-tyrosine that is supplemented to the culture medium, into melanin in liquid cultures. In a recent development, an *E. coli* strain was modified by metabolic engineering to transform glucose to melanin by increasing its capacity to synthesize the precursor l-tyrosine and simultaneously expressing the gene encoding tyrosinase from *R. etli* [14].

**Fig. 1:**
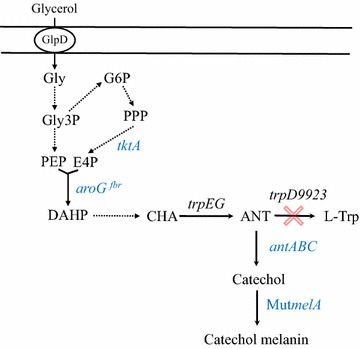
Metabolic pathways related to anthranilate, catechol and melanin biosynthesis in recombinant *Escherichia coli* W3110 *trpD9923*. *Dashed arrows* indicate two or more enzyme reactions. Genes in *blue colour* were overexpressed from plasmids (*aroG* ^*fbr*^, *tktA* and *antABC*) or the chromosome (Mut*melA*). *GlpD* glycerol transporter, *Gly* glycerol, *Gly3P* glyceraldehyde-3-phosphate, *G6P* glucose-6-phosphate, *PPP* pentose phosphate pathway, *E4P* d-erythrose 4-phosphate, *PEP* phosphoenolpyruvate, *DAHP* 3-deoxy-d-*arabino*-heptulosonate 7-phosphate, *CHA* chorismate, *l*-*Trp* l-tryptophan

Most efforts directed towards the biotechnological production of melanins have been directed to eumelanin. This is motivated in part because humans produce eumelanin. However, other types of melanins have physicochemical properties different from those of eumelanin and thus have particular potential applications. The allomelanins are polymers derived from catechol or other substrates. A recent study has demonstrated that a melanin synthesized by the basidiomycetous fungus *Rhizoctonia solani* is derived from catechol [15]. In another report, the production of catechol melanin by the obligate aerobe nitrogen-fixing bacterium *Azotobacter chroococcum* was demonstrated [16]. These organisms could potentially be employed as producers of catechol melanin in a biotechnological process. However, important challenges to overcome would be related to defining the optimal growth and catechol melanin production conditions. An alternative to this approach could be to employ an organism that can be easily grown in a bioreactor under laboratory conditions and genetically modify it to synthesize melanin. In this report, we used the bacterium *E. coli* as a platform to generate strains with the capacity to synthesize catechol melanin. As an initial approach, the Mut*melA* gene from *R. etli* was expressed in *E. coli* W3110 to construct a strain for converting catechol supplemented in the culture medium to catechol melanin. To improve this production system, we employed *E. coli* strain W3110 *trpD9923*, a mutant that overproduces anthranilate, which was modified to produce catechol from glucose [17]. This strain was further engineered by the chromosomal insertion of gene Mut*melA* from *R. etli* (Fig. 1). The resultant strain was grown in liquid fed batch cultures in complex medium with glycerol as carbon source, where 1.21 g/L of catechol melanin were produced in 55 h. The chemical identity of the produced catechol melanin was determined by employing Fourier transform infrared spectroscopy.

## Methods

### Bacterial strains and plasmids

The *E. coli* strains and plasmids employed in this study are described in Table 1. *E. coli* W3110 is a wild type strain, used as a parental host [18]. Strain W3110 *trpD9923* is a mutant in the *trpD* gene generated with ultraviolet radiation, it accumulates anthranilate, and it is an l-tryptophan auxotroph [19]. Plasmid pTrcMut*melA* carries the Mut*melA* gene that encodes a tyrosinase from *R. etli* CFN42 [12]. MutMelA is a mutant version of the MelA tyrosinase, having a single nucleotide change that resulted in the replacement of Asp535 by Gly. It has been determined that melanin production occurs earlier in cultures with *E. coli* strains expressing Mut*melA* when compared to strains expressing wild-type MelA, but melanin rate of synthesis is similar to either version of the enzyme [13]. Plasmid pTrc-*ant3* carries the *antABC* genes coding for the anthranilate 1,2-dioxygenase from *P. aeruginosa* PAO1 [17]. Plasmid pJLB*aroG*^*fbr*^*tktA* carries the *aroG*^*fbr*^ and *tktA* genes from *E. coli* encoding a feedback inhibition resistant version of 3-deoxy-d-*arabino*-heptulosonate 7-phosphate synthase and transketolase, respectively [20].

**Table 1:** *E. coli* strains and plasmids used in this study

Strains	Relevant features	Source
*Strains* W3110	F^−^, λ^−^, INV (*rnnD*-*rnnE*)1	ATCC27325
W3110 *trpD9923*	W3110 [F- Δ- INV (*rrnD*-*rrnE*) 1] *trpD9923* tryptophan auxotroph	[19]
W3110 *trpD9923* Mut*melA*	W3110 *trpD9923* with *R. etli* Mut*melA* gene inserted in the chromosomal *lacZ* locus	This work
*Plasmids*		
pTrcMut*melA*	Gene Mut*melA* cloned in expression vector pTrc99A	[12]
pTrc-*ant3*	Carries the *antABC* genes from *P. aeruginosa* PAO1 under control of the *trc* promoter from pTrc99A, ampicillin resistance	[17]
pJLB*aroG* ^fbr^ *tkt*A pLoxGen*trc*	Carries the *E. coli tktA* gene with its native promoter and the *aroG* ^fbr^ gene under control of the *lacUV5* promoter, tetracycline resistance, pACYC184 origin of replication. Expression-chromosomal integration vector containing the *trc* promoter, a multiple cloning site, the T1 and T2 *rrnB* terminator sequences and the *lacI* ^*q*^ gene	[20] [21]
pLoxGen*trc*Mut*melA*	Derivative of pLoxGen*trc* containing gene Mut*melA* from *R. etli*	This work

### Construction of pLoxGentrcMut*melA* and chromosomal integration of Mut*melA* in *E. coli* W3110 *trpD9923*

A 1830 bp DNA fragment containing gene Mut*melA*, was obtained by digesting plasmid pTrcMut*melA* with restriction enzymes NcoI and SmaI and ligated to plasmid pLoxGentrc digested with the same restriction enzymes [21]. The resulting plasmid was named pLoxGentrcMut*melA*. Plasmid pLoxGentrcMut*melA* was employed as a PCR template to amplify a 3761 bp product that includes the Mut*melA* gene under transcriptional control of the IPTG-inducible *trc* promoter [21]. Primers employed were trcmelAlacZrvs and trcmelAlacZfwd which include 45 bases with homology to the 5′and 3′ terminal regions of *lacZ*, respectively [21]. The PCR reaction was performed using standard buffer conditions, Kapa HiFi DNA polymerase (Kapa BioSystems), one cycle of initial denaturation at 94 °C for 3 min, 30 cycles with denaturing at 94 °C for 30 s, annealing at 56 °C for 60 s and extension at 68 °C for 60 s/kb in a total volume of 50 μL. The PCR product was gel-purified and digested with restriction enzyme DraI to eliminate residual plasmid pLoxGentrcMut*melA* DNA. PCR DNA from the restriction reaction mixture was purified and introduced into strain W3110 *trpD9923* that was previously transformed with plasmid pKD46 that carries the Red-recombinase genes, and Gm-resistant mutants were selected [22]. Candidate strains were screened by plating them in Luria Bertani (LB) solid medium with X-Gal and IPTG to determine the loss of β-galactosidase since the integration event causes the partial deletion of the *lacZ* gene. Colonies displaying Gm resistance and the lack of β-galactosidase activity were screened by performing PCR with primers trcmelA/lacZrvs-SECmelA and trcmelA/lacZfwd-SEC3melA that bind to integrated gene MutmelA and within chromosomal *lacI* or *lacY* genes [21]. A colony yielding the expected 2000 and 3500 bp PCR products was named strain W3110 *trpD9923* Mut*melA*.

### Cultivation media and growth conditions for catechol melanin production

Strain construction and selection of transformants was performed in LB liquid medium. Antibiotics were used at the following concentrations, per mL: ampicillin (Ap), 200 μg for strains transformed with pTrcMut*melA* or pTrc-*ant3*; Tetracycline (Tc), 30 μg for pJLB*aroG*^fbr^*tktA* and Gentamicin sulphate (Gm) 10 µg/mL for pLoxGentrcMut*melA* or 5 µg/mL Gm for strain W3110 *trpD9923* Mut*melA*. Bioreactor cultures were performed in modified M9 minimal salts medium containing per liter: Na_2_HPO_4_ 6 g, KH_2_PO_4_ 3 g, NaCl 0.5 g, NH_4_Cl 1 g, 800 µL of MgSO_4_ 1 M and 80 µL CaCl_2_ 1 M [23]. This medium was supplemented with 2 g/L of yeast extract (to avoid growth limitation as a result of l-tryptophan auxotrophy in strain W3110 *trpD9923*), 40 g/L of glycerol, 20 μg/mL CuSO_4_ and 0.1 mM IPTG. In experiments for the biotransformation of catechol to catechol melanin, glycerol concentration was 40 g/L, 0.85 g/L of catechol was also added to the medium at the start of the culture and yeast extract was omitted. Cultures were performed in a working volume of 800 mL in 1-L stirred tank bioreactors model ADI 1010 (Applikon, The Netherlands). Bioreactor cultures were started from a strain sample taken from a frozen stock that was placed in tubes with 4 mL of LB with the required antibiotics at 37 °C and 150 rpm agitation. After 12 h, the total volume of the previous culture was placed in a 250 mL flask with 75 mL of LB medium with the required antibiotics for each strain and incubated at 37 °C and 150 rpm. After approximately 12 h, the cultures reached an optical density at 600 nm (OD_600_) of 4.0, and an aliquot was taken to inoculate the bioreactor at a starting OD_600_ of 0.2. For bioconversion experiments, a bioreactor with 800 mL of LB supplemented with 20 μg/mL CuSO_4_, 0.1 mM IPTG and Ap 200 μg/mL was inoculated at a starting OD_600_ of 0.2. When the culture reached an OD_600_ of approximately 6.0, the contents of the bioreactor were transferred to bottles under sterile conditions and centrifuged at 4000 rpm for 4 min. The cell pellet was resuspended in modified M9 minimal salts medium supplemented with 20 g/L of glycerol, 0.3 g/L of catechol, 20 μg/mL CuSO_4_, 0.1 mM IPTG and Ap 200 μg/mL. For all bioreactor experiments, air flow rate was maintained at 1 vvm and agitation varied from 500 to 700 rpm to maintain the level of dissolved oxygen above 20 %. Dissolved oxygen was measured with a polarographic oxygen probe (AppliSens; Applikon, Inc., Foster City, CA, USA). Culture temperature was maintained at 30 °C and pH at 7.0. Catechol melanin was recovered from the culture medium by adjusting the pH to 2.0 with HCl 6 N and maintaining it at 4 °C for 16 h. Afterwards, the culture medium was centrifuged for 6 min ant 8000 rpm. The remaining supernatant was left for another 16 h and centrifuged. The precipitated catechol melanin was washed with distilled water and dried at 45 °C for 24 h.

### Analytical methods

Dry cell weight (DCW) was calculated multiplying the 600 nm absorbance by a previously determined coefficient factor of 0.37 g/L [13]. Samples taken during the cultivation period were centrifuged at 10,000 rpm for 2 min at room temperature. The supernatant was filtered using 0.45 μm syringe filter and stored at −20 °C for subsequent analysis. Glycerol and acetate were determined by high performance liquid chromatography (HPLC) (Waters, Milford, MA, USA), using an Aminex HPX-87H column (300 × 7.8 mm; Bio-Rad, Hercules, CA, USA). Running conditions were 5 mM H_2_SO_4_ as mobile phase, a flow of 0.5 mL/min and temperature of 50 °C. Glycerol detection was performed by refraction index detection and acetate by photodiode array at 210 nm. Catechol and anthranilate were determined from the culture medium and not from biomass since the former is the fraction available for recovery. The method employed was HPLC (Agilent Technologies, Palo Alto, CA, USA) using a Synergy Hydro C18 4 μm column (4.6 × 150 mm, Phenomenex, Torrance, CA, USA); running conditions were 0.1 % trifluoroacetic acid in 20 % methanol as mobile phase, a flow of 0.5 mL/min. Detection was performed by photodiode array at 330 nm for anthranilate and 280 nm for catechol. Melanin samples employed for Fourier transform infrared spectroscopy (FTIR) analyses were obtained from the bioreactor cultures described in this article. FTIR spectra were performed at room temperature using a Bruker Tensor 37 spectrometer with a Platinum ATR Accessory by utilizing a resolution of 1 cm^−1^ and 32 scans per spectrum. The samples were finely ground and placed into the sampling area while getting in touch with the diamond ATR-crystal under pressure application.

## Results and discussion

### Bioconversion production of catechol melanin by an *E. coli* strain expressing MutMelA

**Fig. 2:**
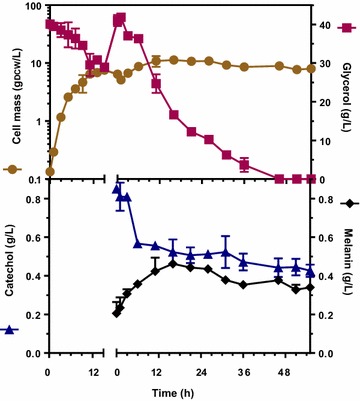
Growth kinetics of inoculum (the first 15 h on the *left side* of the graph) and production cultures (second part of the graph on the *right side*) for bioconversion of catechol to melanin with strain W3110/pTrcMut*melA*. Cell mass (*filled circle*), glycerol (*filled square*), catechol (*filled triangle*) and melanin (*filled diamond*). Graphs represent the mean of three independent experiments

During characterization of an *E. coli* strain that expressed enzyme MutMelA (W3110/pTrcMut*melA*), it was determined that it could employ catechol supplemented to the culture medium as a substrate to generate a dark pigment. This product showed similar characteristics to melanin derived from l-tyrosine, such as low solubility in water and a broad band UV–visible absorption spectrum (data not shown). To determine the capacity of strain *E. coli* W3110/pTrcMut*melA* for generating melanin from catechol, resting cells cultures were implemented. Conditions for obtaining a resting cell culture are usually based on the addition of an antibiotic to arrest cell growth [14]. However, it has been observed that strains expressing MutmelA could not grow on minimal salts medium supplemented with glucose as sole carbon source. When l-tyrosine was supplemented to the medium, the strains displayed growth. These results strongly suggest that MutmelA activity is consuming the endogenously generated l-tyrosine, thus causing a phenotype resembling an auxotrophy [14]. Therefore, under culture conditions that lack l-tyrosine in culture medium, the addition of an antibiotic was not necessary to arrest growth of strain W3110/pTrcMut*melA*. The inoculum for these cultures was prepared in LB medium supplemented with glycerol 40 g/L, where a final biomass concentration of 7.55 g/L was reached after 15 h (Fig. 2). The resting cell cultures were performed in a bioreactor with modified M9 minimal salts medium containing 40 g/L of glycerol and 0.85 g/L of catechol. The initial *E. coli* W3110/pTrcMut*melA* cell mass was 6.44 g_DCW_/L, displaying a small increase during the experiment (52 h). Under these conditions, 0.43 g/L of supplemented catechol were consumed. At the end of the fermentation, cells were removed from culture medium by centrifugation; the supernatant pH was adjusted to 2.0 and maintained at 4 °C for 16 h as described in the Methods sections. By following this procedure, 0.29 ± 0.06 g/L of catechol melanin were recovered from the culture supernatant. Melanin concentration in culture medium has been estimated by several groups by measuring absorbance at 400 nm [11–14]. In this work, we calculated that 1 OD at 400 nm measured at the end of the fermentation, corresponds to 0.12 g/L of melanin recuperated after the precipitation procedure. Figure 2 shows the catechol melanin production profile calculated from absorbance art 400 nm. It coincides with catechol consumption from the start of the resting-cell culture until 15 h. The maximum theoretical yield of melanin from catechol is 1 g/g, therefore, the observed yield corresponded to 67 % of the theoretical maximum.

These results indicate that catechol melanin can be produced in a bioconversion process with *E. coli* W3110/pTrcMut*melA* and also suggest strategies for its improvement. Process productivity can be increased by employing higher DCW and catechol concentrations. It has been determined that catechol at a concentration greater than 2.75 g/L has an adverse effect on strains that produce the aromatic intermediate dehydroshikimic acid [24]. By employing catechol feeding strategies, it should be possible to maintain a catechol concentration below the toxic level during the bioconversion cultures.

### Generation of an *E. coli* strain for the production of catechol melanin from glycerol

Strains with the capacity of producing catechol from simple carbon sources have been reported [17, 24]. This opens the possibility of developing a process for producing catechol melanin from a renewable raw material. Strain *E. coli* W3110 *trpD9923* is a mutant that overproduces anthranilate [19]. This strain was engineered to overproduce this compound by the overexpression of *E. coli* genes *aroG*^*fbr*^ and *tktA*, encoding a feedback inhibition resistant version of 3-deoxy-d-*arabino*-heptulosonate 7-phosphate (DAHP) synthase and transketolase, respectively [25]. Expression of an inhibition-insensitive mutant version of DAHP synthase alleviates feedback inhibition, causing an increase in carbon flow from central metabolism to the common aromatic pathway. The increased level of transketolase caused by plasmid expression of gene *tktA* provides erythrose 4-phosphate (E4P), a direct precursor of DAHP (Fig. 1) [20, 26]. Further modification of this strain by overexpression of genes *antABC* encoding the components of anthranilate 1,2-dioxygenase from *Pseudomonas aeruginosa* PAO1 resulted in an *E. coli* production strain with the capacity to synthesize catechol from glucose [17]. To generate an *E. coli* strain capable of transforming a simple carbon source to catechol melanin, the gene Mut*melA* was integrated into the chromosomal *lacZ* gene of W3110 *trpD9923*, resulting in W3110 *trpD9923* Mut*melA*. This strain was transformed with plasmids pJLB*aroG*^fbr^*tktA* and pTrc-*ant3* to cause an increase in carbon flow from central metabolism to the synthesis of catechol (Fig. 1).

### Bioreactor cultures for catechol melanin production from glycerol with strain W3110 *trpD9923* Mut*melA*/pJLB*aroG*^fbr^*tkt*A/pTrc-*ant3*

**Fig. 3:**
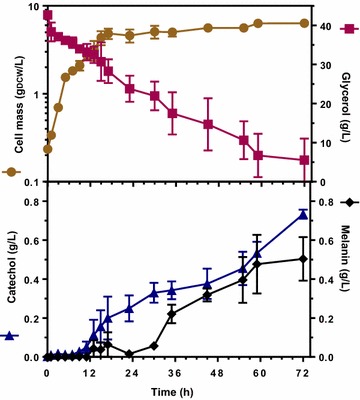
Culture profiles of bioreactor cultures for catechol melanin production from glycerol with strain W3110 *trpD9923* Mut*melA*/pJLB*aroG* ^fbr^ *tkt*A/pTrc-*ant3*. Cell mass (*filled circle*), glycerol (*filled square*), catechol (*filled triangle*) and melanin (*filled diamond*). Graphs represent the mean of three independent experiments

To determine the capacity of strain W3110 *trpD9923* Mut*melA*/pJLB*aroG*^fbr^*tkt*A/pTrc-*ant3* for synthesizing catechol melanin, bioreactor cultures were performed with glycerol as carbon source. Glycerol was chosen since it is an abundant and low-cost raw material that is derived from biodiesel production [27]. An advantage of using glycerol as carbon source when compared to glucose is based on its mechanism of internalization. In *E. coli*, the phosphoenolpyruvate:sugar phosphotransferase system consumes a mol of aromatics precursor PEP for each mol of imported glucose [20]. As consequence, this transport mechanism limits the yield of aromatics production. In contrast, glycerol internalization does not consume PEP, so a higher aromatics yield can be expected when compared to the use of glucose as raw material. In these cultures, yeast extract was also employed, since strain W3110 trpD9923 is an l-tryptophan auxotroph. Batch cultures were performed with strain W3110 *trpD9923* Mut*melA*/pJLB*aroG*^fbr^*tkt*A/pTrc-*ant3* in modified M9 medium supplemented with 40 g/L glycerol and 2 g/L yeast extract. Under these conditions, a growth phase was observed until approximately the 17th h, when the culture entered the stationary phase until the end of the culture at 72 h (Fig. 3). In these experiments, 35 g/L of glycerol were consumed. The accumulation of catechol was observed starting at 12 h until the end of the culture, where the titer was 0.73 g/L. The accumulation of melanin was observed, as evidenced by a change to a dark colour of the culture medium starting around the 18th h, closely coinciding with the start of the stationary phase (data not shown). The amount of melanin recuperated at the end of the culture after precipitation from the total culture supernatant corresponded to a concentration of 1.21 ± 0.24 g/L. The catechol melanin production profile was calculated from the absorbance at 400 nm of culture samples (Fig. 3). It can be observed that catechol melanin concentration at the end of the culture corresponded to 0.5 g/L. This value is lower than the 1.21 g/L recuperated after precipitation of the culture supernatant. This discrepancy can be explained considering that melanin formation depends on the non-enzymatic oxidation of precursors generated by the tyrosinase activity. Some of these precursors are likely not detected at an OD of 400 nm. However, when the culture supernatant is subject to a process that involves a total of 32 h of acid precipitation, this allows for a more complete oxidation of melanin precursors. Therefore, it can be assumed that a significant amount of the melanin recuperated after the pH adjustment process is not detected at 400 nm. These results indicate that gravimetric determination of melanin should be favoured over its quantification by absorbance. It should be noted that the process of melanin formation by microbial cultures is still very poorly understood. It remains to be determined how melanin precursors exit the cell as well as the kinetics of the steps leading to the generation of the aromatic polymer. This process is further complicated by the simultaneous existence of diverse molecular weight forms of melanin, each with distinct physical and chemical characteristics.

It is not possible to provide and accurate estimation of the catechol melanin yield from glycerol since this substrate is being consumed for both cell maintenance during the stationary phase and catechol synthesis. Yeast extract (2 g/L) was added to the culture medium in these experiments since strain W3110 *trpD9923* Mut*melA* is a l-tryptophan auxotroph. To determine if yeast extract was contributing with precursors for melanin formation, a control culture was performed with strain W3110/pTrcMut*melA* under the same conditions employed for melanin production from glycerol. It was determined that 0.06 ± 0.01 g/L of melanin were produced under these conditions. These results indicate that approximately 95 % of the generated melanin is derived from catechol in the cultures with strain W3110 *trpD9923* Mut*melA*/pJLB*aroG*^fbr^*tkt*A/pTrc-*ant3*.

The accumulation of catechol in these cultures indicates that its rate of production surpasses the rate of consumption by tyrosinase (Fig. 3). These data clearly point to tyrosinase activity as an important target for strain optimization. Potential improvement of the production strain could be achieved by exploring gene expression and mRNA optimizations strategies based on synthetic biology approaches. Also, a gene encoding one of the tyrosinases from organisms that naturally produce melanin derived from catechol could be expressed and evaluated in these *E. coli* strains with the aim of improving catechol melanin productivity and titer [15, 16].

### Characterization of the produced melanin

**Fig. 4:**
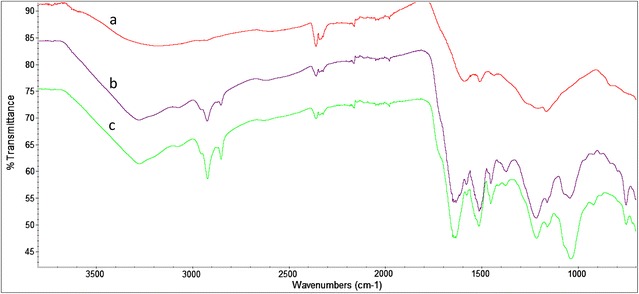
Fourier transform infrared spectroscopy spectra of melanin samples produced by engineered *E. coli* strains from l-tyrosine (**a**), glycerol-derived catechol (**b**) and supplemented catechol (**c**)

IR spectra were determined from melanin samples produced from (1) l-tyrosine (eumelanin) by previously reported recombinant *E. coli* strain [14], (2) glycerol-derived catechol by strain W3110 *trpD9923* Mut*melA*/pJLB*aroG*^fbr^*tkt*A/pTrc-*ant3* and (3) supplemented catechol by strain W3110/pTrcMut*melA* (Fig. 4). A broad band at 3200–3400 cm^−1^ is evident for all samples, although for catechol melanins generated by bioconversion or total synthesis from glycerol, a band at 3274 cm^−1^ is more visible. It relates to stretching vibrations from—OH and –NH groups. Other evident differences can be noticed from the melanin samples produced from catechol or glycerol, compared against the l-tyrosine-derived melanin spectrum, such as the presence of two bands located at 2924 and 2853 cm^−1^, assigned to the stretching vibration of aliphatic –CH, along with a 1454 cm^−1^ band attributed to C–H bending. Chen et al. suggest that these bands, absent in spectrum a, are related to a characteristic difference between l-tyrosine or L-DOPA, and catechol melanins, which are known to be present in *Rhizotocnia* and *Ustilago* species [15]. Also noticeable for samples b and c are the intense bands at 1644 cm^−1^ due to aromatic C=C and –COO– vibrations and the peaks at 1515 cm^−1^ and 1216 cm^−1^, corresponding to C=O stretching and OH deformation in carboxyl groups. All of these are absent in l-tyrosine or l-DOPA-derived melanin. These results show that the two melanin samples, generated from supplemented catechol or from catechol synthesized in vivo by the metabolically engineered production strain, show the main expected absorbance peaks of functional groups for a catechol polymer.

## Conclusions

Melanins are natural polymers having a broad range of potential applications. However, their widespread utilization is limited by the availability of a well-characterized product obtained at a relatively low cost. This is not the case for eumelanin since several microbial strains and biotechnological processes for its industrial production have been reported [11–14]. In contrast, the number of production strains and processes for other types of melanins is very limited. There is only one report on the production in a liquid medium of a melanin derived from homogentisic acid (pyomelanin). By employing the mutant *Pseudomonas putida* strain F6-HDO, 0.35 g/L of pyomelanin was produced [30]. In the case of allomelanins, this report constitutes the first example towards employing a microbial strain developed by metabolic engineering for the gram-level production of catechol melanin from glycerol. These efforts will contribute to a better understanding of the distinct properties of various melanins, leading to potentially unique industrial applications.

## Abbreviations

Ap: ampicillin
Tc: tetracycline
Gm: gentamicin sulphate
LB: Luria–Bertani
OD_600_: optical density at 600 nm
HPLC: high performance liquid chromatography
DCW: dry cell weight
FTIR: Fourier transform infrared spectroscopy
GlpD: glycerol transporter
Gly: glycerol
Gly3P: glyceraldehyde-3-phosphate
G6P: glucose-6-phosphate
PPP: pentose phosphate pathway
E4P: d-erythrose 4-phosphate
PEP: phosphoenolpyruvate
DAHP: 3-deoxy-d-arabino-heptulosonate 7-phosphate
CHA: chorismate
l-Trp: l-tryptophan
